# First report of a newly-described lungworm, *Dictyocaulus cervi* (Nematoda: Trichostrongyloidea), in moose (*Alces alces*) in central Europe

**DOI:** 10.1016/j.ijppaw.2020.11.007

**Published:** 2020-11-29

**Authors:** Katarzyna Filip-Hutsch, Aleksander W. Demiaszkiewicz, Anita Chęcińska, Tomasz Hutsch, Michał Czopowicz, Anna M. Pyziel

**Affiliations:** a Witold Stefański Institute of Parasitology PAS, Twarda 51/55, 00-818, Warsaw, Poland; b Veterinary Diagnostic Laboratory ALAB bioscience, Krucza 13, 05-090, Rybie, Poland; c Molecular Biology Unit, Mossakowski Medical Research Centre PAS, A. Pawińskiego 5, 02-106, Warsaw, Poland; d Division of Veterinary Epidemiology and Economics, Institute of Veterinary Medicine, Warsaw University of Life Sciences–SGGW (WULS), Nowoursynowska 159c, 02-776, Warsaw, Poland; e Department of Food Hygiene and Public Health Protection, Institute of Veterinary Medicine, Warsaw University of Life Sciences – SGGW (WULS), Nowoursynowska 159, 02-776, Warsaw, Poland

**Keywords:** *Alces alces*, Dictyocaulosis, Protostrongylidae, Histopathology, Faecal analysis

## Abstract

Lungworms from the genus *Dictyocaulus* are the causative agents of verminous pneumonia in domestic and wild ungulates. Recently, in 2017, a new species was isolated from red deer and described as *Dictyocaulus cervi*; however, little is known about its epidemiology and pathogenicity in other cervids. The aim of our study was to determine the extent of infection with *Dictyocaulus* nematodes in the moose population in Poland.

Parasitological necropsies were performed in 18 moose and 249 faecal samples were analysed. A combination of multiplex PCR and analysis of the partial SSU, cox1 and cyt B regions revealed the presence of *D. cervi* infection in two of the necropsied moose. Histopathological examinations revealed changes, including multiple cross sections of larvae of nematodes in alveoli, massive pulmonary fibrosis, mononuclear cell infiltration and diffuse alveolar damage in the lungs of four animals. The lesions were more pronounced when adult *Dictyocaulus* nematodes were present in the bronchi and bronchioles. Some of the observed pathological changes could be attributed to co-infection by nematodes of the Protostrongylidae, whose larvae were found in all four animals with lung pathologies. In the faeces, *Dictyocaulus* sp. larvae only occurred together with Protostrongylidae larvae; in addition, higher numbers of Protostrongylidae larvae were excreted in the faeces of animals with dictyocaulosis.

The present study is the first report of the presence of *D. cervi* in moose, and demonstrates the value of multiplex PCR in the identification of *Dictyocaulus* nematodes. Our findings indicate that co-infections with multiple species of lung nematodes in moose may be commonplace, and this should be considered as a factor aggravating the course of parasitosis.

## Highlights

• Newly-described lung nematode *Dictyocaulus cervi* found in moose.
• Verminous and histiolymphocytic pneumonia found with fibrosis and alveolar damage.
• Co-infections between *D.cervi* and Protostrongylid lung nematodes.
• Moose with dictyocaulosis display increased excretion of Protostrongylid larvae.

## Graphical abstract

**Figure:**
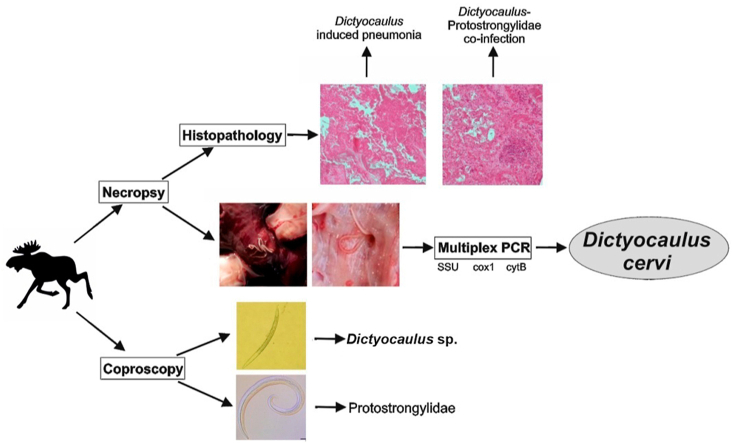

## 1 Introduction

A broad range of domestic and wild ruminants across the Holarctic are subject to bronchopneumonia caused by nematodes of the genus *Dictyocaulus* (Divina et al., 2002; Pyziel et al., 2017). The genus *Dictyocaulus* is represented by several species, including *D. viviparus* isolated from cattle (*Bos taurus*) (Mahmood et al., 2014) and European bison (*Bison bonasus*) (Karbowiak et al., 2014), *D. filaria* found in sheep and goats (Bowman, 2009), as well as *D. capreolus*, characteristic of roe deer (*Capreolus capreolus*) and moose (*Alces alces*) (Gibbons and Höglund, 2002). In addition, a new species named *D. cervi* was recently described in red deer (*Cervus elaphus*) in central Europe and separated from *D. eckerti* (Pyziel et al., 2017); *D. eckerti* had previously been considered the sole nematode characteristic of red deer and reindeer (Skrjabin et al., 1954).

The life cycle of *Dictyocaulus* spp. is direct, with adult nematodes locating themselves in the bronchi and bronchioles in the lungs of the host. First stage larvae hatch from eggs in the airways or large intestine and are excreted with the faeces into the environment. After double moulting, invasive L3 larvae are then ingested by the host while grazing and migrate through the body to reach the lungs as L4.

Infected animals suffer from coughing, increased respiratory rate, loss of appetite and reduced growth (Verstegen et al., 1989; Mahmood et al., 2014). Although dictyocaulosis is known as a cause of high mortality in cattle (Panuska, 2006), it is also common in wild ruminants in Europe, causing severe histopathological changes in the lungs of infected animals (Divina et al., 2002; Pyziel et al., 2018). It is therefore considered a potential threat to the development of the game industry (Gortázar et al., 2006).

The moose is a large mammalian herbivore inhabiting northern Europe, Siberia and north America (Hundertmark and Bowyer, 2004). Exploitation of the species in Poland in the 1970s and 80s resulted in a decline in the moose population by over 70%, and the subsequent imposition of a ban on moose hunting in 2001. Since then, the moose population has increased to almost 28 000 individuals (Wawrzyniak, 2016) and Poland is now considered the southernmost area of moose distribution in Europe (Ratkiewicz et al., 2011). Being heat-sensitive ungulates, moose are particularly exposed to the effects of global warming, including increasing parasite pressure (Borowik et al., 2020). Therefore, it is important to understand the extent of parasitic infections among moose in central Europe to determine their impact on the still relatively fragile moose population.

The aim of our study was to determine the distribution of large lungworm species in the moose population in Poland and to report pathological findings in the lung tissue associated with lungworm infection.

## 2 Materials and methods

### 2.1 Study area

The study was conducted in West Polesie, East Poland and Kampinos Forest, Central Poland.

West Polesie is located on the border with Belarus and Ukraine. In 1990, it was incorporated into the Polesie National Park with an area of 9760.2864 ha, situated in the watershed area of the Bug and Wieprz rivers. It is covered in natural fragments of low, transitory and high peat bogs, mid-forest turf lands and meadow complexes. Almost 95% of the park is occupied by woods, composed of mostly pine and alder trees, while non-woodland areas constitute 5%. Climate is typical for Great Valleys with some continental features, i.e. a long summer and winter. The mean annual air temperature is 7.3 °C. Precipitation varies from 400 mm to 850 mm and most of it falls in the summer (Chmielewski, 2009). According to data from the Polesie National Park Management, it is estimated that about 120 individual moose live in the region, as well as almost the same number of red deer.

Kampinos Forest is situated on the Mazovian Lowland and covers a part of the ancient valley of the Vistula basin. Its area, measuring 38 544.33 ha, is protected by Kampinos National Park, located between the left bank of the Vistula River and the Bzura River, right next to the north-western part of Warsaw. The area of Kampinos National Park is characterized by a combination of sandy dunes and marshes, with dense pine and spruce forest. Almost 73% of the park is occupied by forests, mostly pine. The forest lies in the temperate climate zone and is subjected to transitional marine and continental influences. The mean annual temperature is 7.7 °C, and ranges from 18.6 °C in July to −3.1 °C in January. The mean annual precipitation is 547 mm (Andrzejewska, 2003). It is also estimated that about 300 moose and 330 red deer live in Kampinos National Park.

### 2.2 Collection of lungworms

In the years 2016–2019, five moose killed in road accidents between September and April in West Polesie and 13 moose in the Kampinos Forest were dissected. Respiratory tracts were examined under laboratory conditions. The trachea, bronchi and larger bronchioles were cut open and flushed with tap water. The nematodes were isolated directly from the lumen of the respiratory tract or recovered from the water sediment under stereoscopic microscope. After flushing with tap water, the nematodes were preserved in 70% ethanol.

### 2.3 DNA extraction, amplification and sequencing

Genomic DNA was extracted from the adult lungworms using a Nucleospin Tissue DNA Extraction Kit (Macherey-Nagel, Düren, Germany), according to the manufacturer's protocol. The DNA was isolated from a single nematode from a moose in Kampinos Forest and two nematodes from another moose in West Polesie.

The initial identification of lungworm species was performed by multiplex PCR according to Pyziel et al. (2015). Further classification was performed by amplifying molecular marker genes including small subunit ribosomal RNA (*SSU*), mt cytochrome *c* oxidase subunit 1 (*cox1*) and mt cytochrome *b* (*cytB*). Partial regions of *SSU* and *cox1* were amplified by PCR according to Pyziel et al. (2017) using the following primers: NF50 (5′-TGAAATGGGAACGGCTCAT-3′) and BNR1 (ACCTACAGATACCTTGTTACGAC-3′) for SSU rRNA; COINF (5′-TGTAGATCTATTTCTTTGGARCATAT-3′) and COINR (5′CAGCMCCCAAACTTAAAACA-3′) for cox1. Additionally, a partial region of mt cytB was amplified using the forward primer CytBF (5′-TGAAAARGTTAAGATRRTTGGGAC-3′) and reverse primer CytBR (5′-TTAGGAATAGCACGCAAAATAGC-3′). The primers were designed using FastPCR software, version 5.4 (Primer Digital, Helsinki, Finland) according to Pyziel et al. (2020).

PCR amplification was performed in a 2720 thermal cycler (Applied Biosystems, Foster City, California) in a volume of 50 μl containing the following mix: 20 μl of Molecular Biology Reagent Water (Sigma-Aldrich, USA), 25 μl Quant-Bio's AccuStart™ II PCR ToughMix® (× 2 concentration) (Quantabio, Beverly, USA), 1 μl GelTrack Loading Dye (× 50 concentration) (Quantabio, Beverly, USA), 1 μl forward primer (20 mM), 1 μl reverse primer (20 mM), and 2 μl of template DNA. The thermal profile involved an initial denaturation at 94 °C for 2 min followed by 35 cycles of denaturation at 94 °C (for 40 s for *SSU*, 30 s for *cox 1* and 45 s for *cytB*) annealing (55 °C for 90 s for *SSU*, 58 °C for 30 s for *cox1* and 55 °C for 60 s for *cytB*) and extension at 72 °C (2 min for *SSU*, 30 s for *cox1* and 45 s for *cytB*), with a final extension at 72 °C (10 min for *SSU* and *cytB* and 5 min for *cox1*).

The resulting amplicons were purified with Nucleospin gel and PCR-clean-up kit and eluted with 30 μl of laboratory-pure PCR water. The purified products were sequenced in both directions by Genomed S.A. company (Warsaw, Poland) using the primers given above and additional ones for the SSU rRNA gene: NF890 (CCTAAAGCGAAAGCATTTGCC) and NR1040 (CATACCCCAGGAACCGAA) (Pyziel et al., 2017). The sequences were assembled into contigs using CodonCode Aligner software.

### 2.4 Histopathological examination

Histological assessment was performed on lungs harvested from four moose: two males and two females. Tissue sections fixed in 10% buffered formalin were macroscopically examined and then dehydrated by means of graded ethanol and xylene baths and embedded in paraffin wax. Sections 3, 4 μm wide were cut and then stained with haematoxylin and eosin (HE) and Weigert van Gieson. The microscopic evaluation was performed at 10×, 40× and 100× magnification (objective lens) and photographic documentation was made. The structures of the bronchi, bronchioles, alveoli, pleura and interstitial tissue were examined. The presence of nematode larvae was determined at x40 magnification using an Axiolab A5 light microscope with Axiocam 208 color and ZEN 3.0 software (Zeiss, Jena, Germany).

### 2.5 Faeces collection and analysis

A total of 249 moose faecal samples were collected from January to March in the years 2017–2018 in the area of West Polesie (148 samples) and Kampinos Forest (101 samples). Pseudo replication was avoided by using a sampling strategy that included both temporal and spatial stratification. In most cases, one or two faecal samples were collected from each location.

Three grams of each sample were examined using the Baermann technique (Bowman, 2009) to confirm the presence of lung nematode larvae. Samples were investigated under a stereoscopic microscope (PZO, Poland) at 40× magnification. Larvae were identified to the family or genus level on the basis of morphology (Goliszewska and Demiaszkiewicz, 2007; Bowman, 2009; Lankester et al., 2011).

### 2.6 Statistical analysis

Categorical variables were presented as count and percentage. The values were compared between unpaired groups using the Fisher's exact test, if the expected count in any cell of the contingency table was ≤5, or the maximum likelihood chi-square test if it was not. Comparisons between paired groups were performed using the McNemar's test. In addition, 95% confidence intervals (CI 95%) were calculated using the Wilson score method. Numerical variables were given as median, interquartile range (IQR) and range. The values were compared between unpaired groups using the Mann-Whitney *U* test, and between paired groups using the Wilcoxon signed-rank test. All statistical tests were two-tailed. The significance level (α) was set at 0.05. Statistical analysis was performed in TIBCO Statistica 13.3.0 (TIBCO Software Inc., Palo Alto, CA, USA).

## 3 Results

### 3.1 Macroscopic examination

Gross lesions were observed in the lungs of four out of 18 examined moose: one animal from West Polesie and three individuals from Kampinos Forest. The lungs were airy and pasty and had a soft consistency. Red and white foci were seen on the surface and the edges of lobes. Small (0.5 mm) nodules in the posterior lobes of lungs were detected in one animal from Kampinos Forest.

Twenty adult *Dictyocaulus* sp. nematodes were isolated from the bronchi and bronchioles of a 12-year-old male from West Polesie and another twelve from a two-year-old female in Kampinos Forest. Thus, the prevalence of infection was 11.1% (2/18). The presence of adult nematodes in the bronchi and bronchioles were accompanied by a foamy fluid.

### 3.2 Nucleotide sequences

**Fig. 1:**
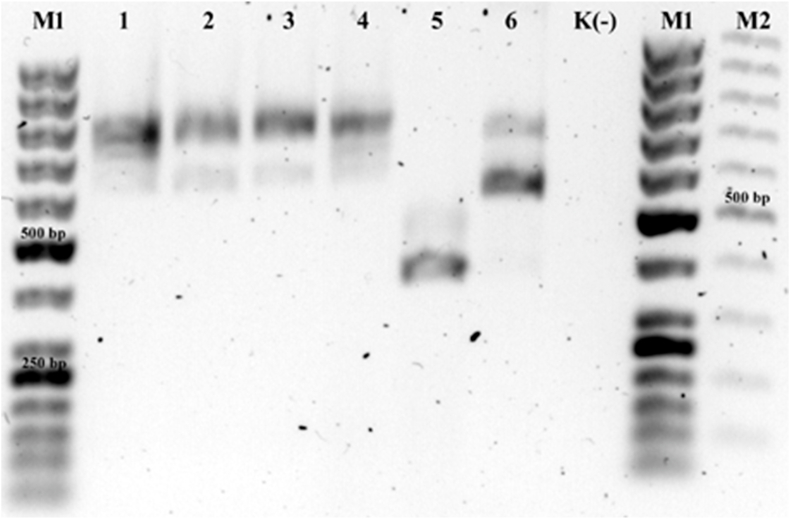
Results of the multiplex PCR test detecting various *Dictyocaulus* species. Lane M1: O'GeneRuler 50 bp DNA Ladder (ThermoFisher Scientific); lane M2: O'GeneRuler 100 bp (ThermoFisger Scientific); lanes 1–4: *D. cervi* product (~800 bp); (lane 1: *D. cervi* from a moose from Kampinos Forest; lane 2: *D. cervi* from a moose from West Polesie; lane 3: *D. cervi* from another moose from West Polesie; lane 4: *D. cervi* from a red deer from north-east Poland); lane 5: *D. capreolus* from a roe deer from north-east Poland (~400 bp); lane 6: *D. viviparus* from European bison from Białowieża Forest (~600 bp); lane K(−): negative control.

The results of the multiplex PCR (Fig. 1) indicated that the examined moose were infected with *D. cervi*: a species that had been previously suspected in red deer and moose in Poland and Sweden (Pyziel et al., 2015), and which represented a newly-established species of red deer lungworm (Pyziel et al., 2017). This was further confirmed by the analyses of nine of the obtained *SSU*, *cox1* and *cytB* sequences (Table 1).

**Table 1:** List of novel sequences obtained in the present study including GenBank accession numbers and primers used.

	West Polesie		Kampinos Forest	
Gene	GenBank	Primers	GenBank	Primers
*SSU*^a^	MT919232	NF50 + BNR1	MT913561	NF50 + BNR1
	MT919231	NF50 + BNR1		
*cox*1	MT914266	COINF + COINR	MT914191	COINF + COINR
	MT914192	COINF + COINR		
*cytB*	MT920217	CytBF + CytBR	MT920216	CytBF + CytBR
	Mt920218	CytBF + CytBR		

a *SSU*, small subunit; rRNA, ribosomal RNA; *cox*1, cytochrome *c* oxidase subunit 1; *cytB*, mt cytochrome *b*.

### 3.3 Histopathological findings

**Fig. 2:**
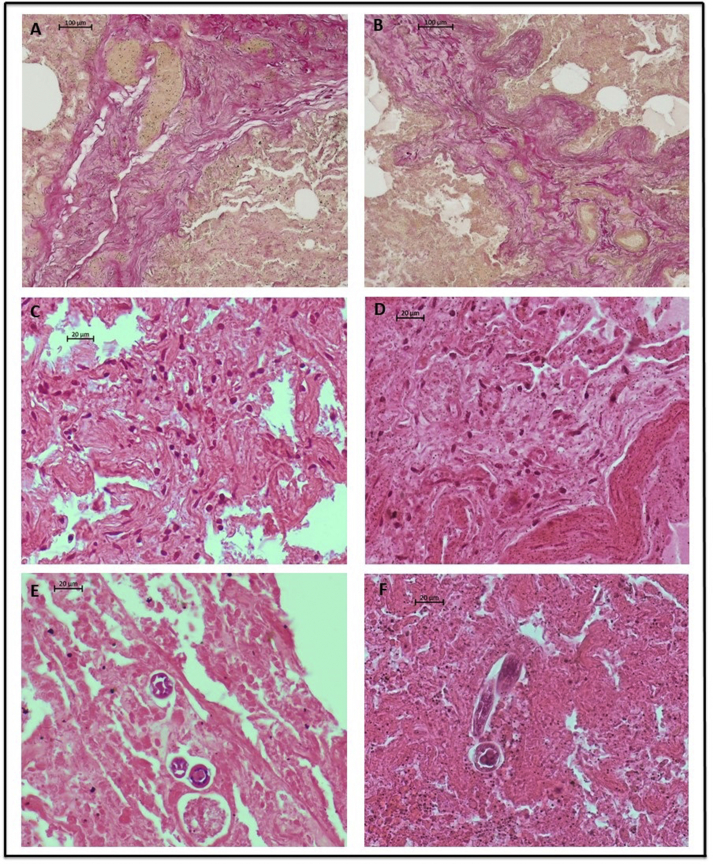
Lung pathology of adult *Dictyocaulus* positive cases. (A and B) Bands of fibrous tissue. Van Gieson's staining, (10× magnification). (C and D) Mononuclear cell infiltration. H-E staining, (40× magnification). (E and F) Cross sections of larvae in alveoli, damaged alveoli. H-E staining, (40× magnification).

**Fig. 3:**
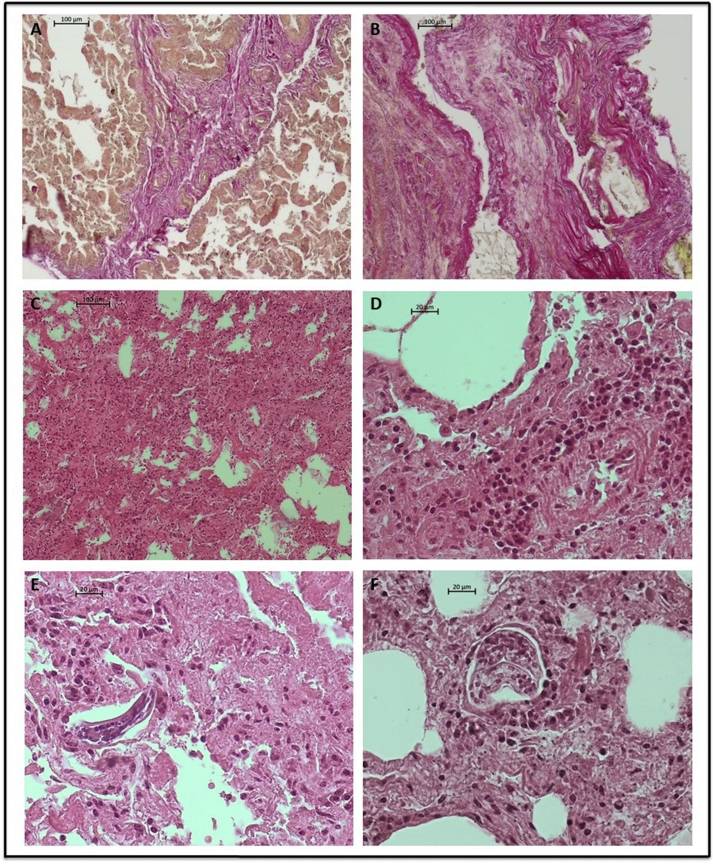
Lung pathology of adult *Dictyocaulus* negative cases. (A) Bands of fibrous tissue. Van Gieson's staining, (10× magnification). (B) Subpleural fibrosis. Van Gieson's staining, (10× magnification). (C) Alveolar damage and mononuclear cell infiltration, exudative fluid located in alveoli and inter-alveolar spaces. H-E staining, (10× magnification). (D) Mononuclear inflammatory infiltration. H-E staining, (40× magnification). (E and F) cross sections of larvae in alveoli, damaged alveoli. H-E staining, (40× magnification).

The lungs of four out of 18 examined moose demonstrated histopathological changes such as larval cross-sections in the alveoli, diffuse alveolar damage and mononuclear cell infiltration (Fig. 2, Fig. 3). Additionally, significant distortions of lung parenchyma caused by severe pulmonary fibrosis were observed; these were characterized by diffuse interstitial and peribronchial foci of connective tissue hyperplasia. In addition, an exudative fluid with foamy appearance was identified in the alveoli and the inter-alveolar spaces. The moose infected with adult *Dictyocaulus* lungworms demonstrated more pronounced lesions in the lungs, while those without adult *Dictyocaulus* demonstrated subpleural fibrosis and nodular lymphoid hyperplasia.

### 3.4 Coprological examination

In total, 249 faecal samples were examined. Both the extensity and intensity of *Dictyocaulus* sp. in the faecal sample were very low and did not differ significantly between West Polesie and Kampinos Forest (Table 2).

**Table 2:** The occurrence of lung nematodes in moose in Kampinos Forest and West Polesie.

	Kampinos Forest (n = 101)	West Polesie (n = 148)	Comparison between Kampinos Forest and West Polesie	Overall (n = 249)
***Dictyocaulus* sp.**				
No. of moose infected	2	3		5
Prevalence (CI 95%)	2.0 (0.5, 6.9)	2.0 (0.7, 5.8)	p > 0.999^a^	2.0 (0.9, 4.6)
No. of larvae in the faecal sample (median, range)	2, 3	1, 3, 4	p > 0.999^c^	3 (1–4)
**Protostrongylidae**				
No. of moose infected	16	39		55
Prevalence (CI 95%)	15.8 (10.0, 24.2)	26.4 (19.9, 34.0)	p = 0.046^b^	22.1 (17.4, 27.6)
No. of larvae in the faecal sample (median, IQR, range)	20, 2–74 (1–270)	21, 6–44 (1–571)	p = 0.578^c^	21, 3–51 (1–571)

a Fisher exact test.

b Maximum likelihood chi-square test.

c Mann-Whitney *U* test.

Although the prevalence of Protostrongylidae larvae was significantly higher in the moose faeces from West Polesie than those in Kampinos Forest (p = 0.046); the intensities in the faecal sample did not differ significantly between the two areas (p = 0.578) (Table 2). .

**Fig. 4:**
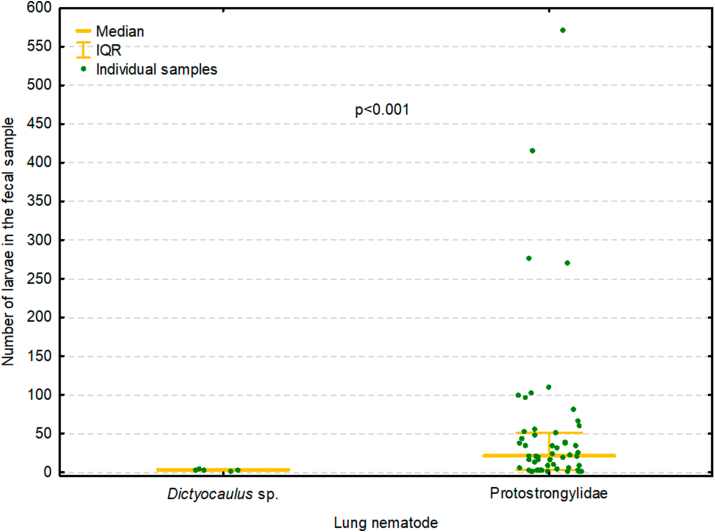
The prevalence of larvae from the genus *Dictyocaulus* and larvae from the family Protostrongylidae in the faeces of moose. IQR – interquartile range.

**Fig. 5:**
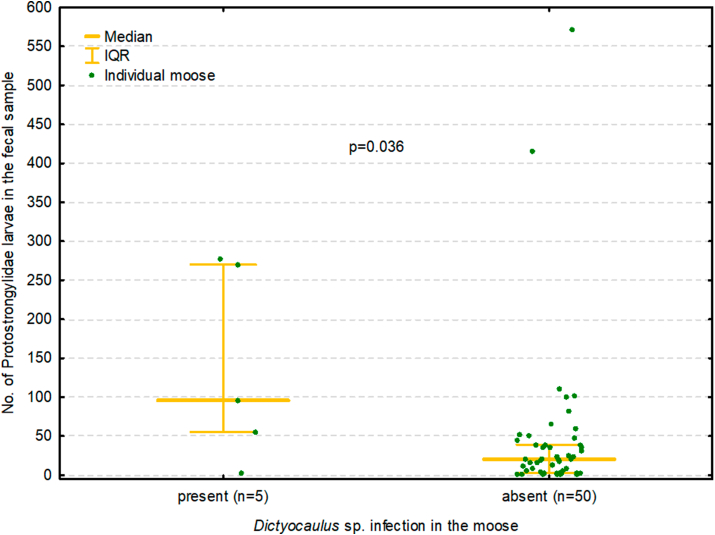
The number of larvae from the family Protostrongylidae in the faecal samples of moose with, and without, larvae from the genus *Dictyocaulus*. IQR – interquartile range.

The prevalence and intensity values of *Dictyocaulus* sp. larvae detected in faecal samples were significantly lower than those of the Protostrongylidae (p < 0.001) (Fig. 4). In addition, *Dictyocaulus* sp. larvae were found only in moose also infected with Protostrongylidae, while the intensity of Protostrongylidae larvae in faecal samples was significantly higher in five moose infected with *Dictyocaulus* sp. (median 96, IQR 55–270) than in 50 uninfected moose (median 21, IQR 3–39) (p = 0.036) (Fig. 5).

## 4 Discussion

Two of the 18 moose necropsied in the present study were found to be infected with *D. cervi* nematodes. The sequencing of the partial *SSU*, *cox1* and *cytB* regions found the identified lungworms to be 100% identical to the *D. cervi* previously isolated from red deer in north-eastern Poland (Pyziel et al., 2017).

Reports of the presence of *Dictyocaulus* lungworms in moose in Europe are very limited. The present study is the first official record of moose infection with *D. cervi*; however, lungworms described as *D. eckerti*, which may have been *D. cervi*, have previously been identified in individual moose in Sweden on more than one occasion (Divina et al., 2000, 2002).

Post-mortem examinations have revealed a similar prevalence of dictyocaulosis between moose in Poland and in Sweden, i.e. about 11%. However, in Sweden, 10.6% of moose were found to be infected with *D. capreolus* and only 0.7% with lungworm, identified as *D. eckerti* (Divina et al., 2002). Although moose in northern Europe are predominantly infected with *D. capreolus*, defined as being specific also for roe deer (Gibbons and Höglund, 2002), this species was not found in the present study.

*Dictyocaulus cervi* is considered as a typical nematode of red deer (Pyziel et al., 2017), and as such, dictyocaulosis is significantly more prevalent in red deer than moose in both central and northern Europe. Lungworms identified as *D. eckerti* were found in 33.3% of red deer in Sweden (Divina et al., 2002); however, there have been no such observations of this species in red deer in Poland. Dróżdż (1966) report that about 70% of red deer in Poland were infected with *D. viviparus*, which was mistakenly considered as cosmopolitan species for all cervids. Recent studies indicate a high prevalence of newly-described *D. cervi* in red deer, reaching levels of 44.6% and 68.2% in north-eastern Poland (Pyziel et al., 2017, 2018). It is possible that co-occurrence of moose and red deer in the studied areas favour transmission of some parasite species, including *D. cervi*, which might be able to persist in the moose population, even in the absence of red deer.

Although the lungs of four moose demonstrated severe histopathological changes, the lesions were more pronounced in the two animals infected with adult *Dictyocaulus* lungworms. The verminous pneumonia with mononuclear infiltration, thickened alveolar septa, exudative fluid in the alveoli or alveolar spaces and massive pulmonary fibrosis (Fig. 2) observed in the present study could be associated with the presence of adult *Dictyocaulus* nematodes in the airways (Panuska, 2006; Pyziel et al., 2018); these symptoms should be considered as characteristic of dictyocaulosis in wild ruminants (Munro and Hunter, 1985; Nashiruddullah et al., 2007; Pyziel et al., 2018).

During histopathological analysis, cross-sections of different developmental stages of lungworms were encountered in the alveoli of all four examined moose, despite the fact that adult *Dictyocaulus* lungworms were only present in the airways of two animals. According to Pyziel et al. (2018), the prevalence of dictyocaulosis is often underestimated, and this may be due to the fact that diagnosis is typically based only on the presence of mature nematodes in the bronchi and bronchioles. More exhaustive histopathological examination is needed to confirm the prepatent phase of infection, identify nematode larvae and evaluate the true prevalence of dictyocaulosis (Jenkins et al., 2007). Therefore, it is possible that some of the changes observed only in the lungs of moose without adult *Dictyocaulus*, such as subpleural fibrosis and lymphoid follicle hyperplasia (Fig. 3), might also be a sign of dictyocaulosis, but its prepatent phase; these symptoms probably result from the migration of the *Dictyocaulus* larvae from lymphatic and blood capillaries into the alveolar parenchyma (Panuska 2006; Nashiruddullah et al., 2007).

Alternatively, some of the observed pathological changes might also occur as a result of infection with lungworms from the family Protostrongylidae, e.g. *Elaphostrongylus alces* or *Varestrongylus alces*, found in moose in northern and central Europe (Verocai et al., 2014; Stéen et al., 2016; Filip and Demiaszkiewicz, 2016; Filip-Hutsch et al., 2020). Fibrosis of the pleura and interlobular septa as well as lymphocytic inflammatory infiltration are also considered typical of *V. alces* infection in moose (Petrosyan, 1963; Verocai et al., 2014). Similarly, the fibrotic changes in the lung parenchyma together with mononuclear infiltration and hyperplasia of the lymphoid follicles found in the present studies have also been observed in the course of elaphostrongylosis in cervids (Steen et al., 1998; Panayotova-Pencheva and Alexandrov, 2011).

Although Protostrongylidae nematodes were not the subject of the present study, their larvae were present in the faeces of all four investigated moose. A few nodules typical for *Varestrongylus* spp. infection (Handeland and Gibbons, 2001; Verocai et al., 2014) were also noted in the lungs of one of the necropsied animals. Verocai et al. (2014) propose that the presence of synergistic and accumulative detrimental effects in the lungs may be associated with parasitism caused by multiple species of lung nematodes. With this in mind, it is possible that the more pronounced pathological lesions observed in the lungs of moose with adult *D.cervi* were caused by a combination of the patent phase of dictyocaulosis and co-infection with Protostrongylidae lungworms, as reported previously (Simpson and Blake, 2018; Verocai et al., 2014).

To evaluate the epidemiology and general spread of dictyocaulosis in the studied populations, an analysis was performed of 249 moose faeces. The results revealed a low prevalence of *Dictyocaulus* larvae, not exceeding 2% (Table 2), which is consistent with other studies of moose in Poland (Filip-Hutsch et al., 2020). A slightly higher prevalence (7.3%) was found in south-eastern Norway (Davidson et al., 2015). The post-mortem and faecal analyses identified a low prevalence of *Dictyocaulus* nematodes in moose, hardly exceeding 10%, which is similar to levels in roe deer and fallow deer (Divina et al., 2002); in contrast, dictyocaulosis has been diagnosed in 94% of European bison and 54% of red deer (Karbowiak et al., 2014; Handeland et al., 2019).

Additionally, the analysis of moose faeces found *Dictyocaulus* larvae to occur only together with Protostrongylidae larvae, and that moose infected with *Dictyocaulus* sp. also excreted higher numbers of Protostrongylidae larvae (Fig. 5). This is consistent with the results of previous post-mortem examinations and might indicate that mixed lungworm infections may well occur in moose in Poland.

## 5 Conclusions

In conclusion, adult nematodes of *D. cervi*, a recently-described parasite of red deer, were found to be present in the lungs of moose in central and eastern Poland. The infected animals demonstrated severe pathological changes in the airways and lung parenchyma; however, some of these probably resulted from co-infection with nematodes of the Protostrongylidae. In such cases, verminous pneumonia, enhanced by the synergistic effect of mixed parasitic infestation, may worsen the condition of the animal and expose it to other infections (Jenkins et al., 2007). Therefore, further studies are needed to fully determine the prevalence of infection with both *D. cervi* and Protostrongylidae lungworms (*Elaphostrongylus* sp. and *Varestrongylus* sp.) in moose in central Europe and to determine the effect of mixed vermicular infection on lung pathology.

## Funding

This research did not receive any specific grant from funding agencies in the public, commercial, or not-for-profit sectors.

## Declaration of competing interest

None.
